# The Clinical Diagnosis and Management of Long QT Syndrome: Insights from the 2022 ESC Guidelines

**DOI:** 10.31083/j.rcm2406170

**Published:** 2023-06-12

**Authors:** Guangqiang Wang, Hongxia Chu, Na Zhao

**Affiliations:** ^1^Department of Cardiology, The Affiliated Yantai Yuhuangding Hospital of Qingdao University, 264000 Yantai, Shandong, China; ^2^Department of Rheumatology, The Affiliated Yantai Yuhuangding Hospital of Qingdao University, 264000 Yantai, Shandong, China

**Keywords:** long QT syndrome, dignosis, management, guidelines

## Abstract

Long QT syndrome (LQTS) is an uncommon disorder that is characterized by QT prolongation and torsade de pointes leading to sudden cardiac death. It is mainly triggered by adrenergic activation. Since LQTS is rare, it is often underdiagnosed. The updated 2022 European Society of Cardiology (ESC) guidelines aim to define the diagnosis of LQTS and spread its management. However, some unknowns and uncertainties still exist regarding the treatment of LQTS. This commentary is geared to the expansion of clinical applications of drug therapies for different subtypes of LQTS based on the 2022 ESC guidelines.

## 1. Diagnosis

**Fig. 1.**
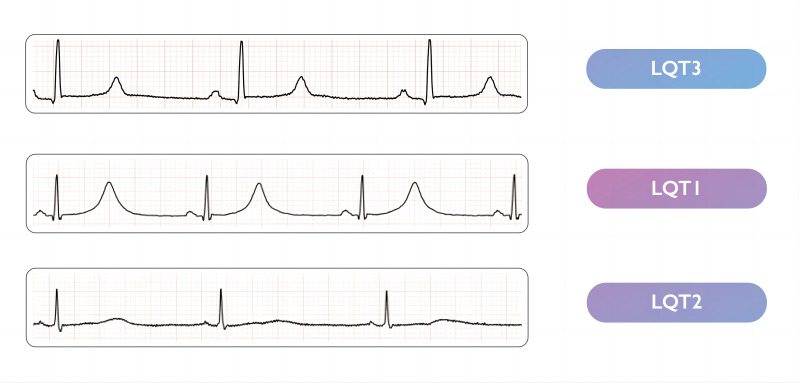
**Electrocardiographic characteristics in the three major phenotypes of congenital long QT syndrome (Obtained from 2022 ESC guidelines [1]. Reprinted by permission of Oxford University Press on behalf of the European Society of Cardiology)**.

We read the updated 2022 European Society of Cardiology (ESC) guidelines on the management of patients with long QT syndrome (LQTS) to prevent the occurrence of life-threatening ventricular arrhythmia (VA) and/or sudden cardiac death (SCD) (Table 1) [1]. LQTS is characterized by QT prolongation and torsade de pointes (TdP), mainly triggered by the activation of adrenergic pathways, including congenital and acquired LQTS. The prevalence of inherited LQTS in the general population was 1:2500. The annual rate of SCD is approximately 5% in symptomatic patients with LQTS [1]. Furthermore, the 10-year mortality rate in symptomatic patients with congenital LQTS is estimated to be approximately 50% [2]. Since LQTS is uncommon, it is often underdiagnosed. There are 17 gene mutations associated with LQTS. Some controversies exist regarding the association of several rare genes with LQTS. It is clear that *KCNQ1*, *KCNH2*, and *SCN5A* account for 75% of clinically definite LQTS cases, whereas 90% of positive genotype cases, and are the main genes causing LQT1, LQT2, and LQT3 triggered by exercise, emotional stress, and sleep, respectively. The majority of LQT1–3 patients present with characteristic electrocardiographic (ECG) ST segment and T wave patterns that prolong the QT interval, each corresponding to a different genotype. Recognition of these gene-specific patterns can increase diagnostic accuracy. LQT3 patients often exhibit a noticeable ST segment prolongation and a distinctive late-appearing normal T wave, whereas LQT1 and LQT2 display a prolonged, broad-based T wave and a low-amplitude notched T wave in the ECG, respectively (Fig. 1, Ref. [1]). A pathogenic mutation was identified in 75% of the LQTS cases by genetic screening, and the remaining cases involving uncertain variants were diagnosed as acquired LQTS.

**Table 1.** **Recommendations for the management of patients with long QT syndrome (Obtained from 2022 ESC guidelines [1])**.

Recommendations		${\mathrm{Class}}^{a}$	${\mathrm{Level}}^{b}$
Diagnosis			
	It is recommended that LQTS is diagnosed with either QTc ≥480 ms in repeated 12-lead ECGs with or without symptoms or LQTS diagnostic score >3.	I	C
	In patients with clinically diagnosed LQTS, genetic testing and genetic counselling are recommended.	I	C
	It is recommended that LQTS is diagnosed in the presence of a pathogenic mutation, irrespective of the QT duration.	I	C
General recommendations to prevent SCD			
	Beta-blockers, ideally non-selective beta-blockers (nadolol or propranolol), are recommended in LQTS patients with documented QT interval prolongation, to reduce risk of arrhythmic events.	I	B
	Mexiletine is indicated in LQT3 patients with a prolonged QT interval.	I	C
Risk stratification, prevention of SCD and treatment of VA			
	ICD implantation is recommended in patients with LQTS who are symptomatic while receiving beta-blockers and genotype-specific therapies.	I	C
	LCSD is indicated in patients with symptomatic LQTS when: (a) ICD therapy is contraindicated or declined; (b) patient is on beta-blockers and genotype-specific drugs with an ICD and experiences multiple shocks or syncope due to VA.	I	C

ECG, electrocardiogram; ICD, implantable cardioverter defibrillator; LCSD, left cardiac sympathetic denervation; LQTS, long QT syndrome; SCD, sudden cardiac death; VA, ventricular arrhythmia. ^a^Class of recommendation. Class I, Evidence and/or general agreement that a given treatment or procedure is beneficial, useful, effective. ^b^Level of evidence. Level of evidence B, data derived from a single randomized clinical trial or large non-randomized studies; Level of evidence C, consensus of opinion of the experts and/or small studies, retrospective studies, registries.

These guidelines reconfirm the previous diagnostic criteria for LQTS: a corrected QT interval (QTc) ≥480 ms or a risk score >3 [2]. The modified LQTS diagnostic score includes ECG, clinical history, family history and genetic findings; it also contributes to individual risk estimation. QTc ≥480 ms on ECG and pathogenic mutations from genetic findings can solely and independently diagnose LQTS because their respective risk score is >3. More importantly, QTc ≥480 ms and pathogenic mutations are associated with acquired and congenital LQTS, respectively. However, pathogenic mutations are the most important diagnostic criteria for LQTS since carriers of pathogenic mutations with normal QTc intervals comprise a certain proportion of patients with acquired LQTS. Genetic testing is essential to avoid genotype-specific triggers, accept genotype-specific treatment for different subtypes, and undergo genetic counselling.

## 2. Pathogenesis

Most of sodium channels inactivate in less than a millisecond during rapid depolarization. However, a relatively small percentage of the sodium channels, designated as late sodium channels, inactivate at a slower rate, resulting in a persistent influx of sodium ions into the cardiomyocytes during the plateau phase of action potential. The residual current flowing through these channels is defined as late sodium current (INa-L) [3]. The pathophysiological mechanism of INa-L in patients with LQTS remains unclear. The possible mechanism is that inherited and acquired conditions, such as “loss/gain-of-function” mutations, structural heart diseases, QT-prolonging drugs and electrolyte abnormalities, regulate the conformational changes of INa-L between the activated and inactivated states, causing the increased dispersion of ventricular repolarization. Previous findings demonstrated that mexiletine regulated the steady-state inactivation process and induced INa-L to enter an inactive state [3, 4]. The gain-of-function mutations in the sodium channel immediately generate a significant increase in INa-L, resulting in the visible prolongation of the ST segment on ECG, such as in LQT3. The gating state of sodium channels may be regulated by interactions among cardiac ion channels. A clinical study revealed that electrocardiographic markers J-Tpeak, representing a balance between block of the human *Ether-à-go-go*-Related Gene (hERG) encoded potassium channel and INa-L block, and Tpeak-Tend, indicating an imbalanced interaction of multiple ion channels, could be used to confirm multichannel effects [4]. The loss- or gain-of-function mutations in other ion channels interactively produced a modest augmentation of INa-L leading to the overall QT prolongation, mainly including the prolonged ST segment and T wave duration. The case in point is LQT 1 and 2, notably suggesting apparent changes in the amplitude, morphology, and duration of the T waveform on ECG (Fig. 1) [1]. Furthermore, increased INa-L is also present in patients with LQT 4, 9, 10, and 12.

Although the hERG cardiac potassium channel block is a major cause of acquired LQTS, the key role of enhanced INa-L has been gradually recognized in acquired LQTS. A prospective clinical trial on drug-induced LQTS affirmed that inhibition of INa-L could reduce the prolonged QTc interval associated with drug-induced hERG potassium channel block. This study also demonstrated that the J-Tpeak interval comprising the whole ventricular vulnerable period inducing ventricular fibrillation was a malignant ECG sign of INa-L [4]. Badri *et al*. [5] observed hERG-mediated potassium channel block presented in acquired LQTS secondary to various underlying causes. The addition of mexiletine in these patients with aforesaid acquired LQTS could shorten the prolonged QTc interval and prevent TdP recurrence.

## 3. Drug Therapy

The guidelines state that β-blockers are recommended as a stand-alone therapy in all LQTS patients regardless of the heart rate [1]. The effect of β-blockers on heart rate is unknown in LQTS patients with slow heart rates. A recent review highlighted that β-blockers could be harmful in LQTS patients with bradycardia-dependent QTc prolongation [3]. β-blockers are recommended as the first-line drug in all LQTS patients, except those with a very slow heart rate [6]. Non-selective β-blockers nadolol and propranolol have better efficacy in inhibiting adrenergic activation to reduce arrhythmic risk for inherited LQTS. INa-L is significantly enhanced at a slow heart rate, and inhibition of INa-L can shorten bradycardia-dependent QTc prolongation. Currently, mexiletine is the only promising alternative to β-blockers for all LQTS patients with markedly prolonged QTc intervals at a slow heart rate [7]. Furthermore, mexiletine can prevent the recurrence of TdP in refractory LQTS [5, 8].

These guidelines recommend that mexiletine is only to be given to LQT3 patients with a QTc prolongation [1]. The establishment of mexiletine as the anti-arrhythmic drug of choice in patients with LQT3 in the 2022 ESC guidelines owes to its efficacy in suppressing recurrent TdP when other drugs are ineffective. Mexiletine is a sodium channel blocker, which is classified as a Vaughan-Williams class Ib anti-arrhythmic drug [9]. A distinct characteristic of mexiletine is that it can shorten the QT-interval as a result of INa-L block which leads to the decrease of action potential duration. As such, mexiletine also acts as a INa-L blocker and INa-L may be a common pharmacotherapeutic target for most subtypes of LQTS [5]. Mexiletine also shortens the prolonged QTc interval significantly in two-thirds of patients with LQT2 [10]. Studies demonstrated that mexiletine could shorten the QTc prolongations in patients with the three main subtypes of LQTS, and that the shortened QTc interval on ECG was more visible in LQT3 patients than LQT1 and LQT2 patients [10, 11]. Research revealed that mexiletine as a INa-L blocker had a positive effect on LQT3 and also showed a wide range of application for the management of other genotypes of LQTS patients with marked QTc prolongation [12]. A retrospective cohort study of 12 LQT2 patients, with long QTc intervals ranging from 547 to 470 ms, mexiletine shortened the QTc intervals with a mean span of 65 ms. In particular, it showed a mean decrease of 91 ms in eight patients whose QTc shortened by ≥40 ms [10]. A small sample-size clinical study observed that mexiletine shortened the prolonged QTc intervals with a mean cut off of 48 ms and avoided proarrhythmic complications in 16 LQT1/LQT2 patients [11]. The case in point was a young Chinese girl with LQT8, characterized by multisystem disorders resulting from *CACNA1C* mutations. Mexiletine shortened QTc from 584 to 515 ms, blunted QT–RR relationship, and abolished 2:1 atrioventricular block and T wave alternans [13]. It can shorten the prolonged QT interval without widening the QRS duration and elevating the ST segment. It can also suppress spontaneous arrhythmogenic activity-triggered TdP and sympathetic stimulation-induced electrical storms [14].

Mexiletine may be antiarrhythmic, proarrhythmic, or both. The beneficial and harmful effects are closely related to its dosage [3]. At a modest dose, mexiletine can shorten the prolonged QTc interval because of the INa-L block, whereas at a high dose, mexiletine inhibits peak sodium current, resulting in a delay in conductivity and suppression of excitability. The potential to cause bradycardia or (ventricular) tachycardia emerges. Although it can block the peak sodium current at a very high concentration, it can preferentially block INa-L at a clinical concentration. Furthermore, it was fairly well tolerated because of its few and mild side effects. Caution should be exercised when using mexiletine in patients with LQTS with critical illnesses.

Although the effectiveness of mexiletine in most LQTS patients is positive, not all LQTS patients can be protected owing to mexiletine-insensitive mutations or undefined electrophysiological properties [3]. Additionally, pharmacokinetic and metabolic factors of mexiletine and other modulators may also play a critical role in the response to mexiletine. The long-term efficacy and safety of mexiletine in LQTS needs further investigation.

The guidelines do not indicate whether mexiletine monotherapy should be administered as a stand-alone therapy or whether the combination of a β-blocker and mexiletine is more effective for LQTS. However, they recommend that the QTc shortened by ≥40 ms on ECG be verified to be effective by performing oral testing [1]. It provides a simple and feasible way for clinicians to verify the effectiveness of drug therapy. Previous studies, combined with our clinical experience with a mexiletine-treated patient with LQT1, suggested that non-selective β-blockers (nadolol or propranolol) concomitant with mexiletine were superior to stand-alone β-blocker therapy in patients with LQT1 and 2 at a high risk of arrhythmic events [15, 16]. A prospective, multicentric, large-scale randomized control trial of mexiletine monotherapy, stand-alone therapy, and combination therapy should be considered to establish the precise role of mexiletine and conclude the most effective therapy for different subtypes of LQTS. The search for new and other potential genotype-specific therapies is ongoing.

## 4. Non-Drug Therapy

These guidelines also provide a lifesaving recommendation of non-drug therapeutic strategies for the treatment of VA and prevention of SCD when pharmacotherapy fails [1]. Use of implantable cardiac defibrillators (ICD) is only recommended in patients with symptomatic LQTS while receiving β-blockers and genotype-specific drugs. ICD discharge can stimulate sympathetic activation, leading to electrical storms. The effectiveness of ICD is certain; however, the tolerance to ICD is not as good as that to drugs. In addition, left cardiac sympathetic denervation (LCSD) is applicable to symptomatic LQTS patients with contraindications or intolerance to ICD implantation. This does not mean that LCSD is an alternative option to ICD in patients with LQTS at high arrhythmic risk. Importantly, LCSD reduces arrhythmic events, as confirmed by only small sample-size studies. The efficacy and safety of LCSD are uncertain in large-scale populations with LQTS. Therefore, drug therapy may be superior to ICD or LCSD treatment, excluding the aforementioned necessary conditions. According to the guidelines, drug therapy is the first choice, followed by ICD implantation for LQTS, and LCSD is limited to auxiliary therapy.

## 5. Conclusions

The guidelines state that genetic analysis of LQTS is crucial for diagnosis, prescription of genotype-specific drugs, and risk stratification. Mexiletine can be applied to different subtypes of LQTS, and concomitant β-blockers may be superior to monotherapies. Pharmacotherapy is recommended as the first-line therapy for LQTS, while ICD implantation is a complementary and alternative therapy. Further large-scale investigations are urgently needed to explore the expanded application of guideline-indicated drug therapies for LQTS.
